# Nutritional supplement practices of professional Ugandan athletes: a cross-sectional study

**DOI:** 10.1186/s12970-017-0198-3

**Published:** 2017-11-13

**Authors:** Haruna Muwonge, Robert Zavuga, Peninnah Aligawesa Kabenge, Timothy Makubuya

**Affiliations:** 1 Department of Physiology, School of Biomedical Sciences, College of Health Sciences, Makerere University, P.O Box 7072, Kampala, Uganda; 2 Habib Medical School, Islamic University in Uganda (IUIU), Kampala, Uganda; 3 Uganda Olympic Committee (UOC), Heskethbell Road, Plot 2-10, Lugogo Sports Complex, P.O.Box 2610, Kampala, Uganda; 4 Uganda Peoples Defense Forces (UPDF), Kampala, Uganda; 5 Department of Sports and Recreation, Makerere University, P.O.Box 7062, Kampala, Uganda; 6 Department of Educator Preparation, Innovation and Research, College of Education, University of Missouri- St. Louis, 364 Marillac Hall, One University Blvd. St. Louis, Missouri, MO 63121 USA

**Keywords:** Uganda, Nutrition, Dietary supplements, Doping, Sport, Athletes, Prevalence, Perceptions

## Abstract

**Background:**

The use of nutritional supplements (NS) places athletes at great risk for inadvertent doping. Due to the paucity of data on supplement use, this study aimed to determine the proportion of Ugandan athletes using nutritional supplements and to investigate the athletes’ motivation to use these supplements.

**Methods:**

A cross-sectional study was conducted in which an interviewer-administered questionnaire was used to collect data from 359 professional athletes participating in individual (boxing, cycling, athletics) and team (basketball, rugby, football, netball, and volleyball) sports. The data were categorized, and a Chi-square test was used for statistical analysis.

**Results:**

Of the 359 athletes, 48 (13.4%) used nutritional supplements. Carbohydrate supplements, energy drinks, vitamin and mineral supplements, fish oils, and protein supplements were the most common supplements used by athletes. NS use was significantly more common among athletes who played rugby and basketball (*X* ^*2*^ = 61.101, *p* < 0.0001), athletes who had played the sport for 5-10 years (*X* ^*2*^ = 7.460, *p* = 0.024), and athletes who had attained a tertiary education (*X* ^*2*^ = 33.377, *p* < 0.0001). The athletes’ occupation had no bearing on whether they used supplements. Nutritionists/dieticians, retail stores and pharmacies were the most common sources of NS products, whereas health practitioners, online media and teammates were the most common sources of information regarding NS. Most athletes used NS to improve their physical performance and health.

**Conclusions:**

Compared to NS use by athletes elsewhere, NS use among Ugandan athletes was low. However, determinants of athlete NS use in the current study (category of sport and duration of time spent playing the sport) are similar to those reported elsewhere.

## Background

The use of doping substances/methods threatens the legitimacy of sports. In an era of round-the-clock media coverage for sporting events and a time where elite athletes bask in the spotlight as social media and television celebrities while earning large sums of money from endorsements in the process, athletes are increasingly under pressure to live up to the hype by winning games at all costs. Despite adopting robust doping detection methods and enforcing severe punishments for doping offenses, athletes continue to use doping agents. A 2015 review on the prevalence of doping in elite sports estimated that over 14-39% of elite athletes intentionally use prohibited substances/methods [1]. Whereas some athletes resort to doping agents as a means of getting an edge over their opponents, a few others, who prefer to operate within the legal boundaries, opt for nutritional/dietary supplements instead.

A dietary supplement is defined as a product that is consumed for the purpose of supplementing the diet and may contain one or more dietary ingredients, including vitamins, minerals, herbs, amino acids and other substances, or their constituents [2]. In many areas, nutritional supplements are marketed as weight loss agents, analgesics, or health, energy, cognitive, and physical performance boosters [3, 4]. However, despite the apparent lack of scientific evidence to support many of their alleged benefits, over 37-98% of athletes continue to use them [5–15]. In turn, this enormous demand among the athlete niche has given birth to multi-billion dollar businesses, which aggressively market the supplements as potent ergogenic aids [3, 4, 16]. It is these vague ambitions that predispose nutritional supplements to unlawful contamination by doping agents. Between 10 and 25% of currently marketed supplements reportedly contain prohibited substances [7, 17]. This contamination has in turn contributed to 6.4-8.8% of the total doping offenses [7], most of them inadvertent.

The most popular supplements consumed by athletes include vitamins and minerals, energy drinks, and protein supplements [5, 6, 8–11]. Performance enhancement, prevention of nutritional deficiencies, better physical appearance, immune system enhancement, and recovery from training and injury are some of the known reasons why athletes use supplements [6, 10, 12, 18]. Additionally, earlier studies identified health professionals, coaches, fellow athletes, the internet, and magazines as the most common sources for information regarding supplements [8, 9, 11].

In Uganda, practitioners of alternative medicine vastly outnumber allopathic doctors, and locally manufactured nutritional supplements, usually packaged as herbal formulations, are marketed as cheaper alternatives to imported nutritional supplements. The proportion of Ugandan athletes who use these supplements is unknown, although unofficial reports indicate that their use is gaining in popularity [19]. As in many other places, the production and marketing of nutritional supplements in Uganda is highly unregulated. Moreover, the active phytochemical substances in most of the preparations are unknown to both the manufacturer and the customer. The fact that most Ugandan athletes are unfamiliar with prohibited doping substances/methods [20] puts them at an additional risk for inadvertent doping through the use of contaminated supplements. In the current study, we determined the motivations to use, perceptions of and prevalence of dietary supplement use among professional Ugandan athletes. The results from this study are intended to help generate recommendations regarding dietary supplement use among athletes living in resource-limited and multicultural environments.

## Methods

### Study design

This was a cross-sectional study involving 359 amateur and professional Ugandan athletes from nine sports: seven major league team sports (football, basketball, football, rugby, boxing, weight lifting and netball) and two individual sports (athletics and cycling).

### Participants

For the study to have >80% power, we used the Kish and Leslie formula for cross-sectional studies to infer a sample size of 339 participants. We anticipated a non-response rate of 5%; thus, we approached 359 athletes to participate in the study. We got a response rate of 100%, as all 359 athletes that were approached consented to take part in the study. Of the 359 athletes, 268 (74.7%) were male, and 91 (25.3%) were female (Table 1). The mean age of the athletes was 25 years, with a large number of participants between 21 and 30 years of age. According to the international standard classification of occupations 2008 (ISCO-08) [21], most of the interviewed participants were either students (20.1%), technicians and associated professionals (19.3%), or service and sale workers (14.8%). Most of the athletes had been participating in the sport for 5-10 years. Approximately 95% of the athletes were presently competing at the national level. On average, we recruited approximately 45 athletes each from volleyball, rugby, netball, basketball, boxing, athletics, cycling, and football. Participants who had retired from a sport or had not participated in a competitive game or competition in the past year were excluded. Stratified random sampling was used in participant recruitment. The independent variables included participant demographic characteristics (age, sex, and level of education), nature of the sport played, duration the sport was played, and the level of competition. The dependent variables included use of nutritional supplements, categories of supplements used, source of supplements, motivation for use of supplements, source of information about supplements, and perceptions about supplement use.

**Table 1:** Socio-demographic characteristics of respondents

Variable	Frequency (n)	Percentage (%)
Sex (359)		
Male	268	74.7
Female	91	25.3
Age (357)		
15-20 yrs	49	13.7
21-25 yrs	144	40.3
26-30 yrs	139	38.9
31-35 yrs	25	7.0
Education level (355)		
Preschool/nursery	2	0.6
Primary school	10	2.8
Secondary school	147	41.4
Tertiary	196	55.2
Occupation (358)		
Manager	9	2.5
Professional	42	11.7
Technician and associate professional	69	19.3
Clerical support worker	31	8.7
Service and sales worker	53	14.8
Skilled agriculture, forestry and fishery worker	16	4.5
Craft and related trades worker	16	4.5
Plant and machine operator	12	3.4
Elementary occupation	32	8.9
Student	72	20.1
Unemployed	6	1.7
Sport played (359)		
Volleyball	46	12.8
Rugby	44	12.3
Netball	45	12.5
Basketball	44	12.3
Boxing	45	12.5
Athletics	45	12.5
Cycling	45	12.5
Football	45	12.5
Duration playing sport (353)		
<5 yrs	86	24.4
5-10 yrs	216	61.2
>10 yrs	51	14.4
Level of competition (359)		
National	340	94.7
International	19	5.3

### Instruments

A pre-tested interviewer-administered questionnaire was used to collect the data. The questionnaire was divided into 2 main sections. The first section captured information on participants’ socio-demographic and sports characteristics such as gender, educational background, occupational history, and sports history (i.e., the type of sport played, the level of competition, and the duration spent playing the sport). The 2^nd^ section consisted of questions about athlete nutritional supplement use, including categories of supplements used, duration and timing of consumption, personal beliefs and motivations for use, and the sources of sports nutrition information. Similar tools have been used previously [22, 23]; [6, 24].

### Procedure

The procedure for data collection that was used in the current study has been used before [20]. Briefly, after guaranteeing complete anonymity and seeking written informed consent from the athletes, a trained research assistant interviewed the participants individually. The interviews were usually held at the teams’ club premises or the training grounds before or after training sessions. After data collection, data were entered into Microsoft Excel 1997-2003 software and then exported to IBM©SPSS© Statistics; version 24 for analysis. Data are presented as frequencies and percentages in tables and charts.

### Statistical analysis

Statistical analysis was performed using IBM©SPSS© Statistics, version 24 (IBM Corporation, New York). Most of the data collected for the different study variables were categorical. Continuous variables, such as athletes’ age and time spent playing a sport were also categorized into groups. After assessing for normality of the data, categorical variables were summarized and presented as frequencies and percentages. Chi-square was used to compare the different categorical variables. A *p* value < 0.05 was considered statistically significant. Permission to perform the study was obtained from the Institutional Review Board of the School of Biomedical Sciences, College of Health Sciences, Makerere University.

## Results

### Athlete characteristics and nutritional supplement use

**Fig. 1:**
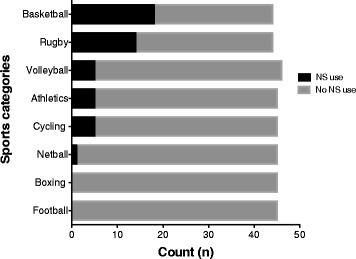
Supplement use among different sports

Athlete response rate in the current study was 100%. Table 2 shows the relationship between athlete supplement use and athlete background characteristics. As illustrated, 48 (13.4%) of the 359 athletes enrolled in the current study used nutritional supplements. Gender was not a significant determinant for supplement use in the current study, with almost an equal proportion of male (12.7%) and female (15.4%) athletes reporting supplement use. Participants of different age ranges were recruited to ascertain whether age was a determining factor for supplement use. We found no significant relationship between age and supplement use among athletes in the current study (*X* ^*2*^ = 2.753, *p* = 0.431). However, the nature of the sport played was a determining factor for supplement use, with more basketball (18) and rugby (14) players reporting supplement use than volleyball (5), athletics (5), cycling (5), netball (1), boxing (0), or football (0) participants (Table 2 and Fig. 1). Moreover, the relationship between the nature of the sport played and nutritional supplement use was statistically significant (*X* ^*2*^ = 61.101, *p* <0.0001). A majority of the athletes (79%) who used supplements had been actively playing or competing in the sport for a period of 5-10 years. The remainder (21%) had competed for either more than 10 years or less than 5 years. The relationship between length of time competing and supplement use was statistically significant (*X* ^*2*^ =7.460, *p* =0.024). Regarding socio-economic status, most of the athletes (45 out of 48) who used supplements in the current study had attained a tertiary education. This relationship was also statistically significant (*X* ^*2*^ =33.377, *p* <0.0001). However, a participant’s occupation had no bearing on whether they used nutritional supplements in the present study (*X* ^*2*^=18.294, *p* =0.075).

**Table 2:** Relationship between supplement use and subject characteristics

Variable	Use supplements?		*P* value	Chi-Square
	Yes (n)	No (n)		
Gender	48	311	0.635	0.427
Male	34	234		
Female	14	77		
Age range	48	357	0.431	2.753
15-20 yrs	3	49		
21-25 yrs	20	144		
26-30 yrs	21	139		
31-35 yrs	4	25		
Occupation	48	311	0.075	18.294
Manager	2	7		
Professional	9	33		
Technician associate professional	5	64		
Clerical support worker	10	21		
Service & sales worker	8	45		
Skilled agriculture, forestry & fishery worker	2	14		
Craft & related trades worker	1	15		
Plant & machine operator	1	11		
Elementary occupation	2	30		
Student	7	65		
Unemployed	1	5		
Sport played	48	311	<0.0001	61.101
Volleyball	5	41		
Rugby	14	30		
Netball	1	44		
Basketball	18	26		
Boxing	0	45		
Athletics	5	40		
Cycling	5	40		
Football	0	45		
Duration playing sport	47	306	0.024	7.460
<5 yrs	5	81		
5-10 yrs	37	179		
>10 yrs	5	46		
Level of competition	48	311	0.750	0.101
National	45	295		
International	3	16		
Education level	48	307	<0.0001	33.377
Preschool/nursery	0	2		
Primary school	0	10		
Secondary school	3	144		
Tertiary	45	151

### Variety, source and intake frequency of nutritional supplements used by athletes

**Fig. 2:**
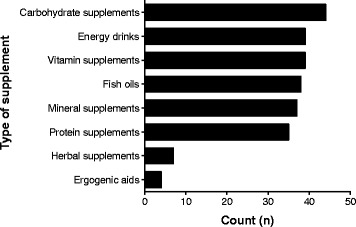
Nutritional supplements used by athletes

**Fig. 3:**
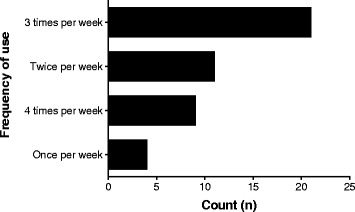
Frequency of supplement use

**Fig. 4:**
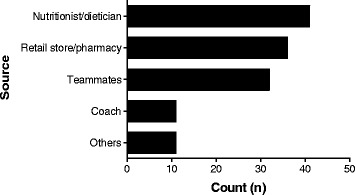
Sources of nutritional supplements

As shown in Fig. 2, the most common supplements used by athletes in the current study were carbohydrates, vitamins, mineral and protein supplements, energy drinks, and fish oils. Surprisingly, few athletes used herbal supplements in the current study. Most of the supplement users took their supplements 3 times per week on average (Fig. 3), and the most common suppliers for the supplements were nutritionists, the local pharmacy, or fellow athletes (Fig. 4).

### Source of nutritional supplement information and athlete’s motivation for supplement use

**Fig. 5:**
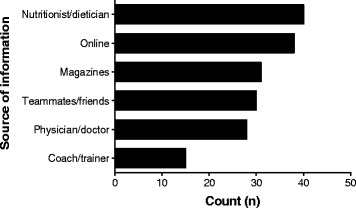
Source of supplement information

The nutritionist/dietician was the most common source of information regarding nutritional supplements, closely followed by online sources, including webpages and social media (Fig. 5). A substantial number of athletes also reported receiving information regarding supplements from their physicians, magazines, teammates, and friends. Interestingly, very few athletes reported receiving information regarding supplements from coaches or trainers.

**Fig. 6:**
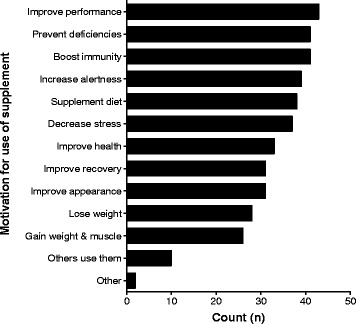
Motivations for supplement use

Most of the athletes used supplements to improve their performance, boost their body’s immune system, prevent deficiencies, and supplement their diets (Fig. 6). Other athletes reported improved health, improved recovery and physical appearance, decreased stress, and increased weight loss as their motivation for supplement use. Very few athletes were motivated to use supplements because other athletes were using them.

### Athlete perceptions about nutritional supplements

**Fig. 7:**
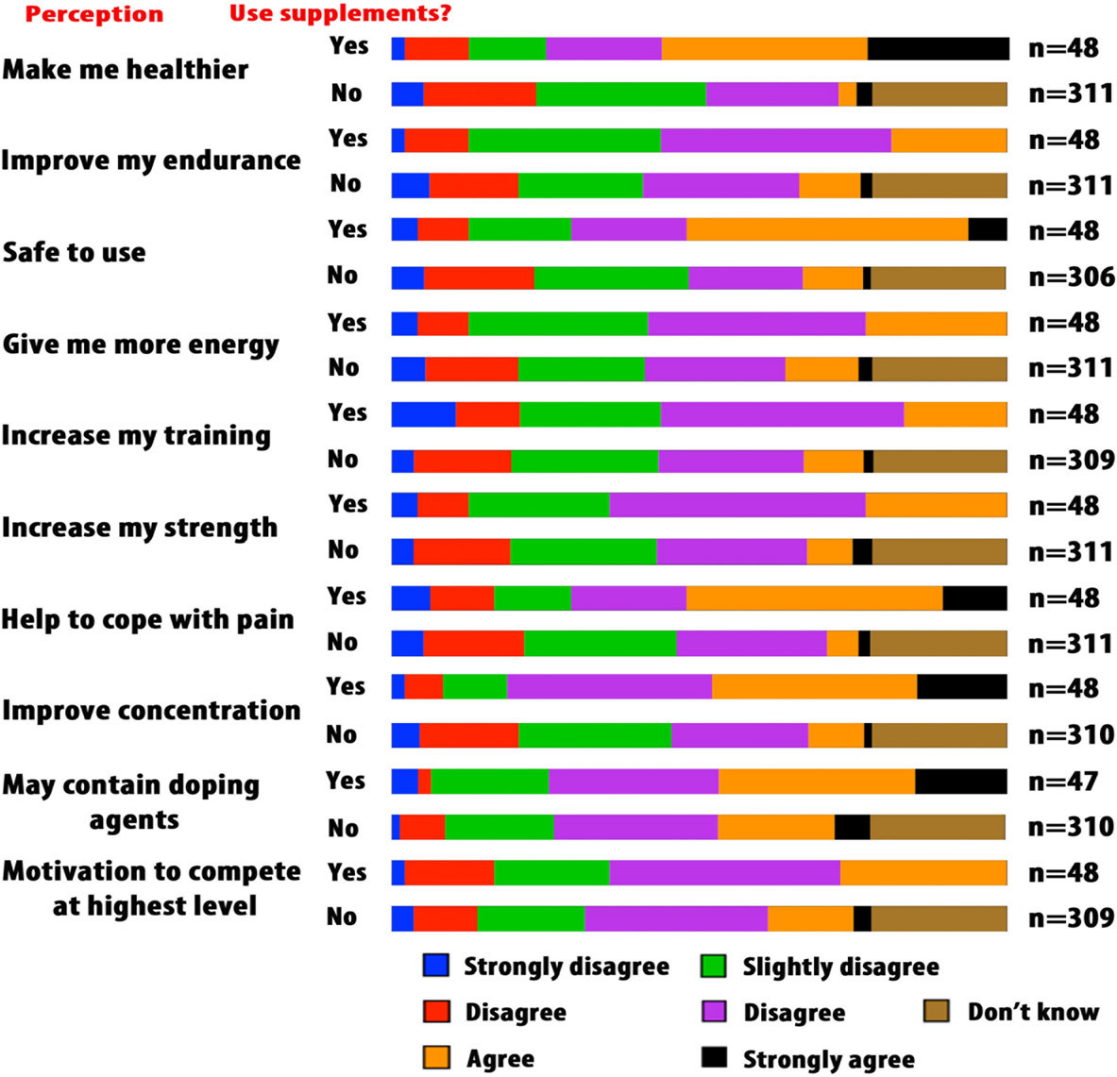
Supplement use and its relationship with athlete perceptions

Athletes were asked for their views on various statements regarding nutritional supplements. These were captured using an agreement scale, ranging from “strongly disagree” to “strongly agree”. An option (“don’t know”) was also provided for those who did not have an answer. The frequencies of the different responses to the statements are shown in Table 3. Figure 7 also shows the athletes’ perceptions regarding different statements about supplement use. Generally, athletes’ opinions concerning nutritional supplement use varied. Most athletes agreed with statements about supplements improving their endurance, increasing their energy and strength, and containing doping agents. In contrast, most athletes disagreed with statements asserting that supplements could improve their health, increase their ability to cope with pain, enhance their concentration during a sporting activity, or were safe to use. The differences observed in athletes’ perceptions to the different statements regarding nutritional supplements were statistically significant (Table 3).

**Table 3:** Athlete perceptions about nutritional supplements

Perception (supplements…)	Strongly disagree (n)	Disagree (n)	Slightly disagree (n)	Slightly agree (n)	Agree (n)	Strongly agree (n)	Don’t know (n)
Make me healthier (n=359)*	17	62	92	76	25	19	68
Improve my endurance (n=359)*	19	46	68	94	49	15	68
Are safe to use (n=354)*	18	59	85	66	52	7	67
Provide me with more energy (n=359)*	19	47	68	85	54	18	68
Increase the amount of training I can do (n=357)*	11	54	79	84	49	13	67
Increase my strength (n=359)*	13	49	78	87	43	21	68
Increase my ability to cope with pain (n=359)*	19	56	83	85	36	11	69
Improve my concentration (n=358)*	15	53	82	85	44	11	68
May contain doping agents (n=357)*	6	24	64	96	74	25	68
Help me train and compete (n=359)*	11	33	61	101	61	22	68

*P value < 0.0001

## Discussion

Overall, 13.4% of professional Ugandan athletes in our study used nutritional supplements. Most of these athletes played either basketball or rugby. Athletes who used supplements mostly consumed carbohydrate supplements, energy drinks, vitamin and mineral supplements, protein supplements, and fish oils. These supplements were consumed 3 times per week on average, and most supplements were acquired from a dietician, a local retail store, a pharmacy, or from fellow teammates. Most athletes consumed supplements to improve performance, boost immunity, prevent deficiencies, improve physical appearance, and decrease stress. Regarding perceptions of nutritional supplement use, athletes who used supplements believed that the supplements made them healthier, were safe to use, helped them cope with pain, improved their concentration, and contained doping agents. Most athletes who lacked knowledge about supplements had never used them before.

The prevalence of supplement use among professional athletes in the current study is low compared to what was previously reported ([5, 8, 13, 18]; [6, 14]). One explanation for this finding could be that the assessment tools for supplement use adopted by the current study may have underestimated the true prevalence of supplement use in an athlete population. The reported prevalence of supplement use in the current study compared to the prevalence from a similar study involving rugby players under the age of 16 in South Africa [14] is low even when corrected for geographic location and population characteristics. Because of the paucity of data on supplement use in developing countries, many parts of the current study tool were adapted from similar studies performed in developed and middle-income countries. As such, less emphasis was placed on the herbal varieties of nutritional supplements, which are indigenous and popular among the local people in Uganda. This may have resulted in under-reporting of supplement use by the athletes. However, it is still possible that the reported prevalence is a true representation, since no dietary evaluation was undertaken to ascertain that the athletes were nutritionally deficient, and thus required nutrient supplementation.

Socio-economic status is a known determinant of supplement use amongst athletes, with athletes of higher education status, or those having higher level paying jobs reported to use supplements more than their counterparts of lower education status, or those with lower paying jobs [3, 4, 25, 26]. These findings partly conform with findings from our study, where nutritional supplement use was significantly higher in athletes with a tertiary education. However, despite its predictive potential from earlier studies [3, 4, 25, 26], athlete’s occupation in the current study had no significant association with nutritional supplement use. Small numbers of supplement users in the occupational sub-groups in the current study possibly confounded this association.

The prevalence of supplement use by athletes of both genders in the current study was similar. This finding corroborates previous observations [3, 4], although it is not consistently reported in the literature [6, 13]. Unlike in previous research findings [15], the athletes’ age had no correlation with supplement use in the present study. However, the duration of time spent playing the sport was significantly correlated with nutritional supplement use; supplement use was more prevalent among athletes who had competed in the sport for 5-10 years than in their counterparts who had competed for shorter (<5 yrs) or longer durations (>10 yrs). It is proposed that younger athletes (who may share similar characteristics with athletes who have competed for <5 yrs so far) have a lower perceived need for supplementation, because they believe that their diets provide an adequate amount of nutrients [8].

Additionally, patterns of dietary supplement use are known to differ between different sports. A cross-sectional study involving 2783 Greek athletes found dietary supplement intake to be higher in athletes who were performing in individual sports than in athletes who were taking part in team sports. This is in contrast to the findings from the current study, in which more athletes from team sports, such as basketball and rugby, consumed supplements than their counterparts from individual sports, such as cycling, boxing and athletics. Fixture scheduling could be one plausible explanation for the discrepancies in supplement consumption, since athletes from basketball and rugby usually have more calendar fixtures and competitions than cyclists, boxers and track-and-field athletes. This could imply that team players have less time in between competitions to recover, which could motivate athletes to consider other alternatives that could enhance recovery, such as nutritional supplements. Also of interest in the current study is the fact that none of the participants who played the sport of football used supplements. Similar findings were reported when Sri Lankan professional football players were surveyed for supplement use [25]. A lower socio-economic and educational status for most professional footballers compared to athletes from other sports sub-groups could possibly account for this outcome.

The main athlete motivations for the use of supplements in the current study included improving performance, boosting the immune system, preventing deficiencies, and supplementing diet and are similar to the motivations reported in earlier studies [5, 6, 12, 18]. In addition to using supplements for ergogenic and cosmetic reasons, the most frequent motivations reported by athletes in the current study can be categorized as “health related”. This could imply that some athletes probably used nutritional supplements because they thought their diets could not supply adequate nutrients for their body’s needs. Furthermore, Wardenaar et al. [27] evaluated elite and sub- elite athletes in Netherlands to understand the adequacy of their intake of micronutrients. From the results of their study, both users and non- users of sports products reported inadequate in-take of the necessary micronutrients. Diets that are scarce in such nutrients are obviously deleterious to athletes, especially those with a rigorous training and competition schedule. More so, many athletes in impoverished parts Sub- Saharan Africa, like Uganda might already be nutritionally disadvantaged compared to their Dutch counterparts.

Carbohydrate supplements, vitamin and minerals, protein supplements, and fish oils were the most frequently used supplements by athletes in our study. This was consistent with findings from earlier studies [6, 8, 9, 11, 12]. The athletes’ motivation for supplement use is known to influence the choice of supplement that is used [13, 18]. In the present study, the fact that the athletes preferred carbohydrates, vitamins, minerals, fish oils and amino acid supplements could be a direct reflection of their motivations for supplement use: improved performance and health. These data are corroborated by findings from another study on athlete dietary habits, where elite Finnish athletes used vitamin and mineral supplements to prevent nutritional deficiencies [18]. Elsewhere, professional Greek athletes cited endurance improvement as the main reason for using amino acid supplements [13]. Due to the popularity of alternative medicine in Uganda, we expected the prevalence of herbal supplement use by athletes in the current study to be high. An earlier study involving Singaporean University athletes identified herbal preparations as a popular supplement used by athletes [11].The popularity of alternative medicine practice is comparable in both Uganda and Singapore. Surprisingly, herbal supplement use in the current study was low, similar to a report from a Sri Lankan study in which athlete supplement use was low, despite herbal formulations being a popular treatment remedy [25]. In Sri Lanka, just like in Uganda, herbal remedies are used as treatment for illnesses rather than as dietary supplements, a fact that could possibly account for the low levels of intake by athletes in the current study. Nonetheless, considering the risks of contamination with prohibited substances, this could be a key positive finding due to the fact that marketing of herbal supplements is unregulated in Uganda. Moreover, athletes in the current study frequently acquired information regarding supplements from health professionals (nutritionists/dieticians and physicians), media (online sources and magazines), and fellow teammates. With the exception of coaches and trainers (who were the least utilized source for supplement information in the current study), similar sources of information regarding supplements have been reported in earlier studies [6, 8, 9, 11]. Over the past 15 years, internet usage in Uganda has increased by 300-fold, growing from 40,000 users in the year 2000 to over 12 million users in the year 2016 [28]. Most of these users are concentrated in urban areas, which is representative of most of our study respondents. This could explain why online media sources were the most common sources of information in our study, since the internet is a readily accessible outlet containing information about supplements. Additionally, we postulate that some athletes might avoid coaches/trainers due to a recent anti-doping push targeting elite athletes and those seeking to compete at the world stage. Sottas et al (2011) reported that the International Association of Athletic Federations (IAAF) introduced blood testing in 2001 and advocated for strict adherence of the protocol, effective from 2005. We also speculate that athletes in our study believed that their coaches/trainers lacked the required expertise to offer advice on nutritional supplements, and thus, athletes were hesitant to seek information from them. This speculation contrasts with earlier findings where athletes preferentially relied on information sources for which accuracy was not guaranteed [6, 11]. Nonetheless, licensing requirements for coaches/trainers in developing and developed nations are different. The minimum requirements for obtaining a coaching/trainer license in most developing countries are not as stringent as those in middle- and high-income countries. In high-income countries, even though most coaches possess a bachelor’s degree or higher, coaches are additionally required to complete specialized coaching education before they can be certified. The curriculum for this specialized coaching education contains a module about sports nutrition. Some coaches from developed countries even undertake additional nutrition training as part of their continued professional development because they can afford it [29–31]. These extra modules ensure that these coaches are better prepared to address nutritional concerns for their athletes than are their counterparts from developing countries.

Athletes in the current study often obtained their supplements from personal or team nutritionists/dieticians, retail stores, pharmacies, or from teammates, which represents an obvious reflection of the athletes’ preferred sources of information regarding nutritional supplements. Depending on whether an athlete used supplements, the response to different statements regarding supplement use was different (Fig. 7). Generally, athletes who used supplements in the present study thought that supplements made them healthier, were safe to use, helped them cope with pain, improved their concentration, and contained doping agents. Even though the current study used these statements to elicit athlete perceptions of supplement use, these statements have previously been used to provide insight into the perceived awareness of athletes on supplements [6]. Knowledge about supplements is believed to deter athletes from using supplements, and a greater understanding of supplements is associated with diminished use of supplements [32]. In contrast, the majority of athletes in the current study who responded with “I don’t know” to different statements regarding supplement use either were not using at the time or had never used nutritional supplements. However, our findings agree with previous findings [6] showing that British track junior national track and field athletes who used nutritional supplements had greater knowledge levels about supplements.

### Study limitations

The major limitation of the current study is that we may have underestimated the true prevalence of supplement use among athletes. Taking into consideration the popularity of alternative medicine practice in Uganda, the assessment tool that we adapted to collect data on herbal supplements did not capture the broad range of herbal supplement formulations and varieties that are unique and indigenous to Uganda. However, the remaining items on the adapted questionnaire had been standardized in earlier studies [22, 23]; [6, 24]. This facilitated comparisons between our data and data that were generated from earlier studies. Furthermore, due to the small number of supplement users in our study, we were unable to complete more robust statistical analyses to examine further associations between factors among nutritional supplement users. However, the 100% response rate of athletes and the adequate statistical power in our study ensured that a satisfactory comparison was made between athletes who used supplements and those who did not.

Most athletes use supplements because they believe their diets do not adequately meet their daily nutritional needs. We did not assess for the nutrient content of the athletes’ diets, so we cannot speculate on the relationship between athlete supplement use and dietary nutrient adequacy. Designs for future studies should take these factors into consideration.

## Conclusion

To our knowledge, this is the first report of its kind on nutritional supplement use among professional athletes in Uganda. It unequivocally confirms that professional Ugandan athletes readily use nutritional supplements, mainly to maintain their health and to improve athletic performance. However, the reported prevalence of supplement use in the current study is lower than what has been reported in other studies. The category of sport played, the duration of time the sport was played for, and the athletes’ education status were all found to influence dietary supplement use in the current study. Playing basketball or rugby and obtaining a tertiary education were positively associated with dietary supplement use. Online media, health professionals, and fellow athletes were the most common sources of supplement information, and retail stores/pharmacies were the most common sources for purchasing dietary supplement products. Carbohydrate supplements, energy drinks, vitamin and mineral supplements and fish oils were the most commonly used supplements. However, only a very small proportion of athletes used herbal supplements in the present study. Further investigation is required to confirm the nature of this association. Future study tools should include an extensive catalog of herbal supplements indigenous to the local population.

## Abbreviations

ISCO-08: International standard classification of occupations 2008
NS: Nutrition supplements
UOC: Uganda Olympic Committee
